# Advances in High-Voltage Power Electronics Using Ga_2_O_3_-Based HEMT: Modeling

**DOI:** 10.3390/ma18204770

**Published:** 2025-10-17

**Authors:** Reem Alhasani, Hadba Hussain, Mohammed A. Alkhamisah, Abdulrhman Hiazaa, Abdullah Alharbi

**Affiliations:** King Abdulaziz City for Science and Technology (KACST), Energy and Industry Sector, Microelectronics and Semiconductors Institute, Riyadh 11442, Saudi Arabia; malkhamisah@kacst.gov.sa (M.A.A.); ahiazaa@kacst.gov.sa (A.H.)

**Keywords:** gallium oxide (Ga_2_O_3_), high electron mobility transistor (HEMT), two-dimensional electron gas (2DEG), ultra-wide bandgap (UWBG), interface polarization, TCAD simulation, power electronics, GaN substrate, surface charge modeling, threshold voltage

## Abstract

Gallium oxide (Ga_2_O_3_) is a promising ultra-wide-bandgap (UWBG) material with exceptional transport properties, including a large breakdown voltage, making it ideal for high-voltage power device applications. Recently, Ga_2_O_3_ has gained significant attention as a next-generation material for electronic device fabrication aimed at advancing power electronics. In this paper, we investigate the effect of a Ga_2_O_3_ buffer layer on a GaN-based high electron mobility transistor (HEMT), focusing on output I–V characteristics and surface charge effects. Furthermore, we explore an advanced approach to enhance HEMT performance by utilizing polarization-induced two-dimensional electron gas (2DEG), as an alternative to conventional doping methods. A III-N/Ga_2_O_3_ heterostructure is proposed as a distinctive electrical property and a cost-effective UWBG solution. To evaluate the associated effects, we simulate a two-dimensional (2D) Ga_2_O_3_/GaN HEMT structure incorporating surface charge models. Our results confirm that 2DEG formation near the surface creates a conductive channel due to polarization-induced dipoles at the interface. The simulations also show a negative shift in the threshold voltage, a condition typically unattainable without oxidation layers or doping. Finally, we analyze the potential of AlGaN/Ga_2_O_3_-based HEMTs for future power electronic applications.

## 1. Introduction

Gallium oxide (Ga_2_O_3_) has emerged as a highly promising semiconductor material for next-generation power electronic applications, owing to its outstanding intrinsic properties, namely, its ultra-wide bandgap (UWBG) and high breakdown electric field strength [1,2,3]. Early studies of Ga_2_O_3_ primarily centered on its luminescent behavior; however, in recent decades, attention has increasingly turned to its potential for enabling advanced electronic and optoelectronic devices [1]. This shift has been driven by global demands for more efficient, compact, and thermally robust power systems, as well as by significant progress in wide-bandgap semiconductor technologies [1].

The bandgap of Ga_2_O_3_ is typically characterized as ultra-wide, with the most stable β-phase exhibiting a bandgap in the range of approximately 4.7 to 4.9 eV. Nonetheless, the precise value of the bandgap may exhibit slight variations contingent upon the polymorphic form and the degree of crystalline disorder, with certain studies indicating bandgap values as high as 5.1 eV or potentially lower for specific disordered configurations [4,5,6].

Traditional semiconductors, including silicon (Si) and even wide-bandgap materials like silicon carbide (SiC) and gallium nitride (GaN), face inherent limitations under high-voltage and high-temperature conditions [7,8]. Their relatively narrow bandgaps, limited breakdown fields, and modest thermal conductivities result in reduced efficiency and operational reliability under strenuous electrical or thermal loads [7,8]. In addition, the oxide semiconductor materials, such as SrSnO_3_ [9] and SnO_2_ [10], also have limitations, where the latter’s limitations include reduced carrier mobility in thin films, limited dopant solubility, and potential instability [11].

Ga_2_O_3_ provides a compelling alternative due to its ultra-wide bandgap of approximately 4.9 eV, a theoretical breakdown field nearing 8 MV/cm, and excellent chemical and thermal stability [12]. Empirical studies and device-level demonstrations have confirmed its ability to achieve significantly higher breakdown voltages, reduced conduction losses, and improved overall energy efficiency compared to conventional materials [1,13]. These attributes position Ga_2_O_3_ as a transformative material for high-voltage, high-efficiency power conversion systems.

Nonetheless, several material and device-level challenges must be addressed before Ga_2_O_3_ can achieve widespread deployment. One of the most critical limitations is its inherently low thermal conductivity, which hinders effective heat dissipation and imposes reliability concerns during continuous high-power operation [14,15]. To mitigate this issue, recent studies have explored advanced solutions such as thermally conductive composite materials, engineered substrates, and novel doping techniques aimed at enhancing heat transport while maintaining favorable electrical characteristics [14,15].

In parallel, research is advancing fabrication techniques to improve the crystal quality and uniformity of Ga_2_O_3_ layers. Key developments include refined epitaxial growth processes, enhanced doping accuracy, and strategies for suppressing material defects [16,17]. These improvements have proven essential for producing devices with consistent and reproducible performance metrics, which are prerequisites for commercial scalability [16,17]. The development of advanced characterization tools has also played a pivotal role in accelerating Ga_2_O_3_ research. High-resolution microscopy, electrical probing, and spectroscopic methods have enabled a detailed understanding of the relationship between material properties—such as defect density, crystal orientation, and doping profiles—and device performance [18]. These insights have informed the design of more robust device architectures and have facilitated the optimization of fabrication workflows [18].

**Figure 1:**
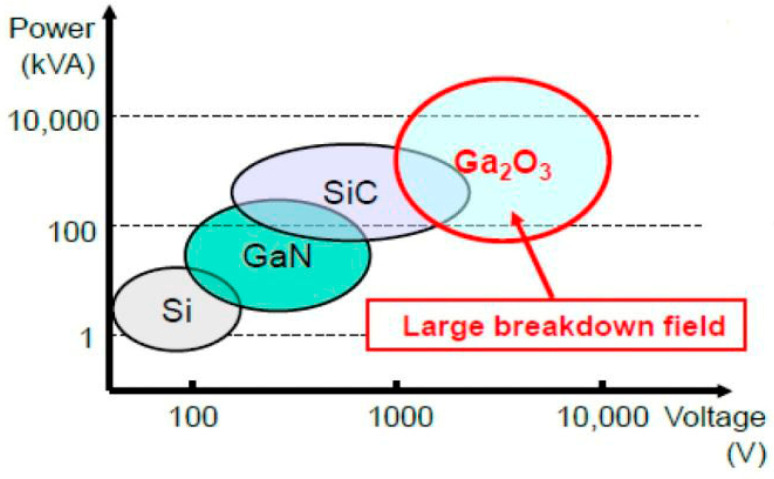
Key of the power-voltage semiconductor UWBG materials of power electronics application.

To contextualize Ga_2_O_3_’s performance, comparative studies with other conventional UWBG materials—namely, GaN, SiC, and diamond [1,19]—have provided important benchmarks [20,21,22]. While Ga_2_O_3_ offers distinct advantages such as the availability of native substrates and higher theoretical breakdown strength, it also lags in areas like thermal conductivity and doping capability. These comparative analyses help guide the design of hybrid systems and inform material selection for specific high-voltage applications [20,21]. Figure 1 power versus voltage application space for key semiconductor materials used in power electronics. Ga_2_O_3_ occupies a unique position due to its exceptionally large breakdown field, enabling it to handle high voltage, high power levels, and high-efficiency applications [22,23], compared to that of conventional semiconductors such as Si and SiC [13,24,25].

High-power electronics, with their electrical properties, support the development of compact, high-voltage power devices that outperform in many applications. Particularly, Schottky barrier diodes (SBDs) fabricated from β-Ga_2_O_3_ provide low forward voltage drop and reverse leakage current, resulting in high efficiency and thermal resilience [26]. Vertical device configurations using bulk β-Ga_2_O_3_ enhance heat dissipation and withstand greater electric stress. Current research focuses on advanced surface passivation and optimized metallization to address these limitations and enhance device performance. Photodetectors and optoelectronics β-Ga_2_O_3_’s strong absorption in the solar-blind deep ultraviolet (DUV) region (<280 nm), combined with its transparency to visible light, makes it highly effective for UV photodetection. This spectral selectivity is crucial in secure communication, flame detection, and environmental monitoring. Innovations such as heterojunction photodetectors combining β-, α-, and ε-phase Ga_2_O_3_ with p-GaN, and in situ GaON interlayers, improve interface quality and charge separation [27].

Ongoing research aims to improve device responsivity, sensitivity, and operational stability in low-power conditions [27,28]. Wafer bonding using in situ silicon thin films offers a viable route for integrating Ga_2_O_3_/Si interfaces without high-temperature annealing. This reduces thermal mismatch and maintains the structural integrity of UWBG and silicon materials [29]. High electron mobility transistors (HEMTs) based on AlN/β-Ga_2_O_3_ grown on SiC substrates demonstrate significant potential for radio frequency and portable power applications. Their UWBG characteristics and superior electron mobility enable high-frequency operation, reduced power loss, and improved heat dissipation. The use of SiC substrates contributes further to thermal stability and mechanical reliability under harsh operating conditions such as those in aerospace and defense [13].

Plasma-Enhanced Chemical Vapor Deposition (PECVD) enables the deposition of amorphous Ga_2_O_3_ thin films at temperatures below 200 °C, allowing integration with flexible polymer substrates. These films exhibit high optical transparency, chemical and thermal stability, and sufficient carrier mobility for use in flexible displays, UV sensors, and wearable electronics [30]. Graphene/β-Ga_2_O_3_ heterojunctions are being explored to improve contact properties in Ga_2_O_3_-based devices, leveraging graphene’s high conductivity, mechanical flexibility, and chemical robustness [31]. These developments are crucial for producing scalable and reliable transparent or wearable electronic systems. In epitaxial growth and substrate engineering, the choice of substrate—such as sapphire, MgO (110), or epi-GaN—affects lattice match, defect density, and carrier transport. To mitigate lattice mismatch, buffer layers and precise growth condition control are applied. Current efforts focus on enhancing crystal uniformity and expanding integration to cost-effective, thermally matched substrates like SiC, with the aim of achieving scalable, high-performance device fabrication [3,32,33].

Calculations based on ab initio have been performed on the vacancy defect of the β-Ga_2_O_3_ crystal [34,35]. Usseinov et al. revealed that in instances where pair vacancies are thermally activated, the transition energy levels are elevated to approximately 2.0 eV above the upper boundary of the valence band. At this energy level, the recombination of electrons and holes becomes feasible, as similarly evidenced in the scenario involving single vacancies [34]. Furthermore, Hao et al. conducted an examination of polarons within β-Ga_2_O_3_. Their findings indicate that small hole polarons may arise at various oxygen sites, specifically, OI, OII, and OIII, with the OII site demonstrating the highest stability. Additionally, there is no indication of the existence of polaronic states for electrons, whether large or small, in β-Ga_2_O_3_ [35].

In a recent study, Xianxu Li et al. successfully synthesized β-Ga_2_O_3_/ε-Ga_2_O_3_ phase junctions on sapphire substrates utilizing an atmospheric-pressure chemical vapor deposition process. Consequently, the phase junction demonstrates a type II energy band alignment, with measured valence and conduction band offsets of 0.54 eV and 0.41 eV, respectively [36]. Moreover, the engineering of Ga_3_O_2_ substrates encompasses the enhancement of material characteristics and device efficacy through growth on indigenous substrates [24] or the application of a buffer layer on non-native substrates [37,38].

Ga_2_O_3_ demonstrates inherently low and anisotropic thermal conductivity, with the beta (β) phase exhibiting values generally varying between 11 and 29.6 W/(m·K), contingent upon the specific crystallographic direction. This characteristic is affected by several determinants, including crystallinity, the quality of interfaces, and the presence of defects, which are crucial for its utilization in power electronics and thermal management applications [37,39,40,41,42].

Furthermore, due to the limited thermal conductivity inherent in β-Ga_2_O_3_, it is essential to implement thermal management strategies at the device scale to facilitate high-power performance. Research conducted by Samuel Kim et al. demonstrates that the integration of double-sided cooling alongside a heat spreader can significantly reduce the thermal resistance of the device from 24.5 to 4.86 mm·°C/W, thereby enabling a high power density [43].

A recent investigation by A. Revathy et al. studied a β-Ga_2_O_3_ substrate with a 55 nm InAlN/InGaN/GaN/AlGaN HEMT, achieving impressive DC characteristics, including a peak drain current density of 5.5 A/mm and an ON-resistance of 9.23 Ω mm. The device displayed excellent electrostatic control with an I_ON_/I_OFF_ ratio over 10^13^ and a maximum transconductance of 0.77 S/mm. Reliability was confirmed with a breakdown voltage of up to 55 V [3].

Recent experimental progress has highlighted the remarkable potential of β-Ga_2_O_3_ for high-power electronics. For example, Cai et al. (2023) demonstrated normally-off β-Ga_2_O_3_ MOSFETs with a threshold voltage of ~9 V and breakdown voltage of 834 V, emphasizing the feasibility of enhancement-mode operation [44]. Similarly, β-Ga_2_O_3_ trench Schottky barrier diodes fabricated by novel Ga-flux etching achieved a record breakdown field of >5.10 MV/cm with a breakdown voltage of 1.45 kV [45]. In 2024, vertical enhancement-mode β-Ga_2_O_3_ FinFETs reported an average breakdown strength of 2.7 MV/cm with positive threshold voltages [46].

The ongoing advancement of Ga_2_O_3_-based power devices is underpinned by multidisciplinary efforts encompassing materials science, device engineering, and system-level integration. The overarching objective remains to realize power electronics that are not only more efficient and compact but also capable of operating under increasingly stringent thermal and electrical conditions. Continued innovation in this field is expected to substantially contribute to the development of more sustainable and high-performance energy technologies [1,12].

In this work, N/Ga_2_O_3_ is proposed as a material with distinctive electrical properties and as a cost-effective option among UWBG materials. Thus, we investigate the effect of a Ga_2_O_3_ buffer layer-based HEMT, focusing on interface charge effects and output I–V characteristics. Furthermore, we explore an advanced process to enhance HEMT performance by utilizing polarization-induced 2DEG due to polarization-induced dipoles at the interface, via simulating a 2D Ga_2_O_3_ HEMT structure incorporating surface charge models using numerical simulation based on a TCAD software [33]. The simulation results suggest high device performance and a shift in the threshold voltage.

Despite these advances, most prior studies rely on experimental demonstrations with specific substrates or device geometries. In contrast, the novelty of this work lies in performing substrate-free TCAD simulations, which provide a direct comparison of Ga_2_O_3_ against other wide-bandgap semiconductors. Our results reveal distinct advantages of Ga_2_O_3_, including its wider bandgap and higher critical electric field, and benchmark the simulated I-V and threshold voltages against the most relevant experimental reports. This establishes Ga_2_O_3_ as a fundamental candidate for future high-power device applications.

## 2. Methodologies

Modeling and simulation of the HEMT device structure, using Ga_2_O_3_ as a buffer layer of device in three cases: without foreign substrate, with GaN substrate, and with SiC substrate.

**Figure 2:**
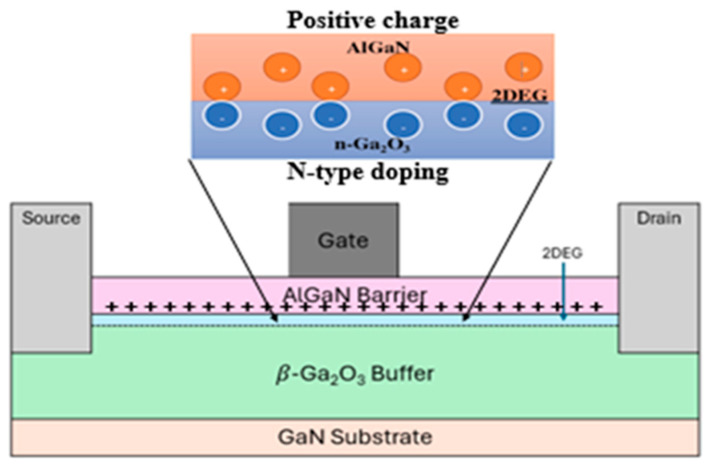
HEMT device structure using β-Ga_2_O_3_ as buffer layer. In AlGaN/n-Ga_2_O_3_ HEMT, a 2DEG is generated at the interface as a consequence of polarization phenomena and interface charge.

**Figure 3:**
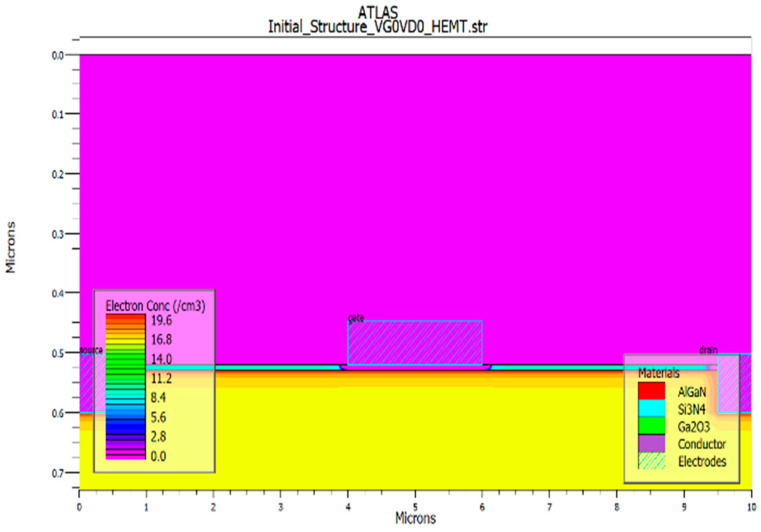
Ga_2_O_3_ HEMT diagram of the heterostructure at zero bias conditions (at V_g_ and V_d_ = 0 V) without foreign substrate, with Q_f_ = 1 × 10^10^ cm^−2^. The 2DEG was confirmed close to surface with high electron concentration.

The high electron mobility transistor (HEMT) device is comprised of a 1200 nm GaN substrate doped with p-type conductivity at a concentration of 1 × 10^17^ cm^−3^, succeeded by a 200 nm buffer layer of β-Ga_2_O_3_ doped with n-type conductivity at a concentration of 1 × 10^18^ cm^−3^. Over the buffer layer, an undoped AlGaN barrier layer measuring 10 nm is placed. The undoped AlGaN (barrier) and doped β-Ga_2_O_3_ (buffer) layers constitute two distinct semiconductors that establish a heterojunction. This heterojunction facilitates the spontaneous formation of a two-dimensional electron gas (2DEG) layer as illustrated in Figure 2. This is achieved through the transfer of electrons from the doped layer to the undoped layer, resulting in the accumulation of charge carriers at the interface, which forms the 2DEG. The defined gate length of the device is 2 μm (L_G_), while the source (L_S_) and drain (L_D_) lengths are 0.5 μm, with a total channel length measuring 9 μm. Ohmic contacts for the source and drain were established by setting the work function of the electrode at 3.93 eV, whereas the Schottky gate contact was achieved by defining the electrode work function at 5 eV. The voltage applied to the drain was maintained at a constant value of 50 V, while the voltage on the gate was incrementally adjusted from −16 V to 16 V in steps of 4 V. Detailed structural parameters of the HEMT device are presented in Table 1. Figure 3 illustrates the schematic cross-sectional design of the HEMT utilizing β-Ga_2_O_3_ as a buffer layer.

TCAD software 2D device simulation.

**Figure 4:**
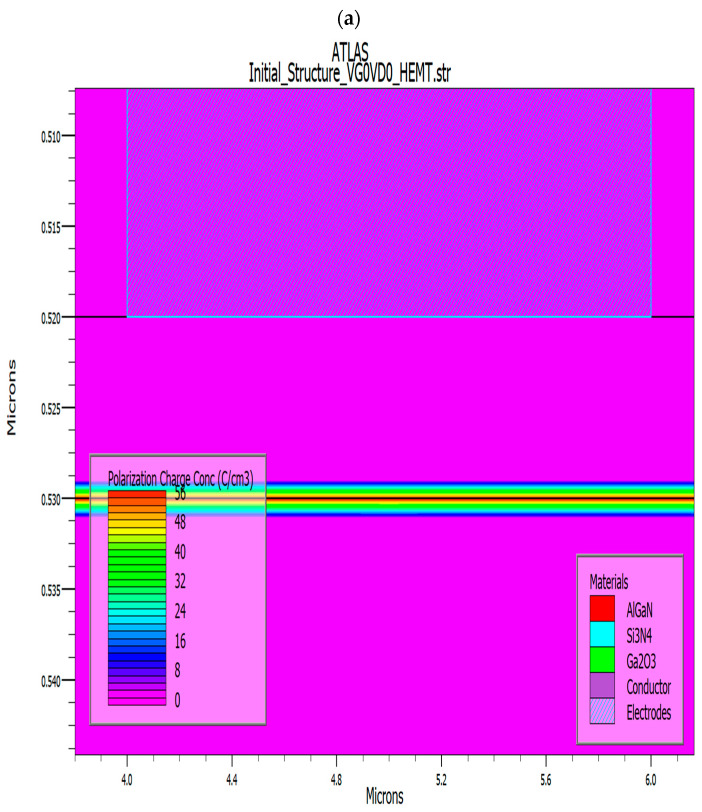

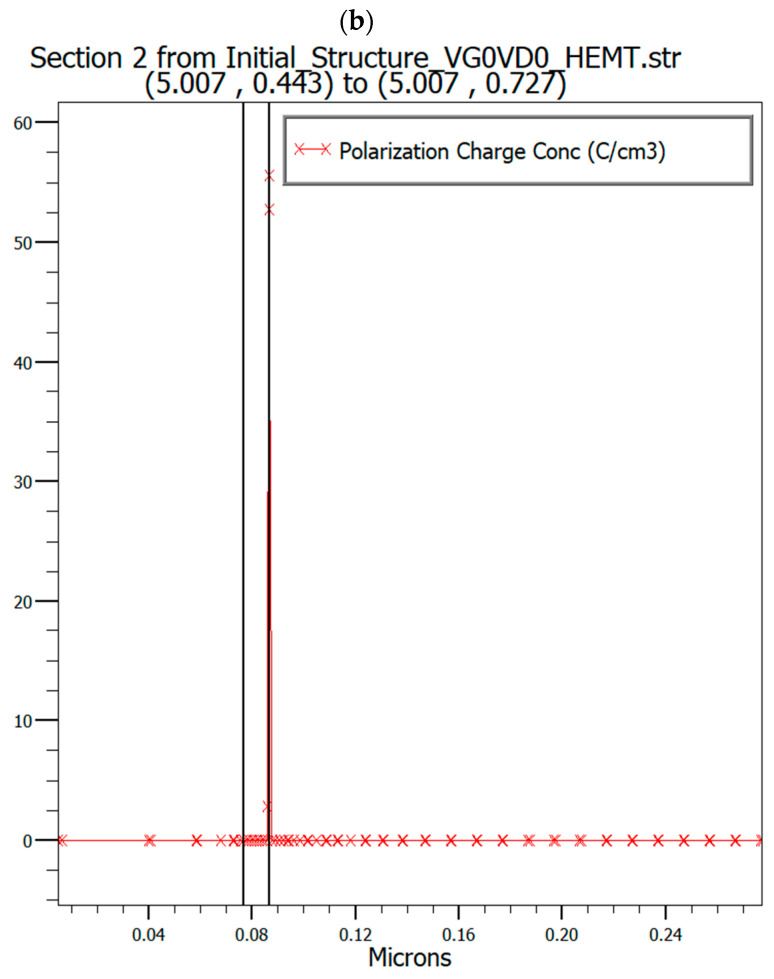
The polarization charges are 55 and its distribution throughout the Ga_2_O_3_ HEMT device. (**a**) Polarization structure and (**b**) polarization interface without substate, with Q_f_ = 1 × 10^10^ cm^−2^, and at V_g_ and V_d_ = 0 V.

The energy band diagram resulting from the TCAD simulation at zero gate bias and drain bias conditions is illustrated in Figure 3. The polarization interface charge in the 2DEG originated from the built-in electric field which generated due to the discontinuity in spontaneous and piezoelectric polarization present between two distinct semiconductor materials (AlGaN/β-Ga_2_O_3_). This electric field facilitates the formation of a 2DEG at the interface by confining electrons from the bulk material into a narrow region, thereby delivering the elevated mobility essential for the operation of HEMT [38]. This diagram reveals the conduction band discontinuity present at the interface of AlGaN/β-Ga_2_O_3_. The sheet charge density (Q_f_) of 1.0 × 10^10^ cm^−2^ was derived from the device simulation. Complementary details regarding the polarization charge are depicted in Figure 4. The material parameters incorporated into the simulation are summarized in Table 2 [47]. Moreover, the device structural details such as the defects, strain, and so on in the device simulation are illustrated in Table 3.

The fundamental device physics models applicable to all semiconductor devices are grounded in Maxwell’s equations, Schrödinger’s equation, the Shockley–Read–Hall (SRH) recombination framework, Poisson’s equations, continuity equations, and the drift-diffusion transport equations [1]. The quantum electron density is determined by the one-dimensional (1D) Schrödinger’s equation, which links the eigenstate energies $E_{iv}$(x) and the wave function $\varphi_{iv}$(x, y). The subsequent sections provide a comprehensive discussion of these equations.

(1) $$-\frac{h^{2}}{2}\frac{\partial }{\partial y}\left(\frac{1}{m_{y}^{v}\left(x,y\right)}\frac{{\partial \varphi }_{iv}}{\partial_{y}}\right)+E_{c}\left(x,y\right)\varphi_{iv}=E_{iv}\varphi_{iv}$$

In this context, $m_{y}^{v}$(***x***, ***y***) represents the spatially dependent effective mass, while $E_{c}$(***x***, ***y***) denotes the conduction band edge. Poisson’s equation serves as a significant partial differential equation that is widely applied within the domains of electrostatics and theoretical physics. In semiconductor modeling, it often provides the essential framework for deriving quantitative solutions for electrostatic parameters [1]. The equations formulated by Poisson delineate a relationship among the electric field (**E**), electrostatic potential (**ψ**), and spatial charge density (***ρ***). This relationship can be articulated as follows [1]:

(2) $$\nabla^{2}\Psi =-\nabla E=\frac{\rho }{\varepsilon }$$

The continuity and transport equations define the temporal changes in electron (n) and hole (p) densities resulting from carrier transport mechanisms, carrier generation processes (designated as *G*_*n*_ and *G*_*p*_), and carrier recombination phenomena (denoted as *R*_*n*_ and *R*_*p*_). The continuity equations are expressed through the subsequent mathematical expressions [1]:

(3) $$\frac{\partial_{n}}{\partial_{t}}=\frac{1}{q}\nabla J_{n}+G_{n}-R_{n}$$

(4) $$\frac{\partial_{p}}{\partial_{t}}=-\frac{1}{q}\nabla J_{p}+G_{p}-R_{p}$$

The drift-diffusion model pertaining to carrier transport is firmly rooted in Boltzmann transport theory, which delineates a relationship between the current densities (*J*_*n*_ and *J*_*p*_) and the quasi-Fermi levels (∅_*n*_ and ∅_*p*_). Additionally, these quasi-Fermi levels are connected to carrier concentrations and potentials through the application of Boltzmann approximations [1]:

(5) $$J_{n}=-q\mu_{n}n\nabla \emptyset_{n}$$

(6) $$J_{p}=-q\mu_{p}n\nabla \emptyset_{p}$$

The application of a gate voltage to the device results in a proportional alteration of the sheet charge concentration n_s_, as articulated in Equation (7). In this equation, $\epsilon_{N}$ is the permittivity of the barrier, ${d=d}_{i}+d_{d}$ is the thickness of the doped-plus-undoped barrier layer, ∆d is a correction factor, q is the elementary charge, $V_{g}$ is the gate voltage, and $V_{off}$ is the threshold voltage [48].

(7) $$n_{s}=\frac{\epsilon_{N}}{q\left(d+∆d\right)}\left(V_{g}-V_{off}\right)$$

The threshold voltage constitutes a pivotal parameter as it indicates the point at which the HEMT device initiates conduction. $V_{off}$ is defined in Equation (8) as:

(8) $$V_{off}=\phi_{B}-\frac{{\Delta E}_{c}}{q}-\frac{qN_{D}}{2\epsilon_{N}}d^{2}$$

where $\phi_{B}$ represents the Schottky barrier height at the gate, ${\Delta E}_{c}$ denotes the alteration in the conduction band at the heterojunction, and $N_{D}$ refers to the background doping level of the β-Ga_2_O_3_ layer [48]. Meanwhile, the saturation current I_DSat_ can be expressed as Equation (9):

(9) $$I_{DSat}=\frac{\epsilon_{N}W}{\left(d+∆d\right)}\left(V_{g}-V_{off}-V_{0}\right)\nu_{sat}$$

where $\nu_{sat}$ is the carrier saturation velocity and $V_{0}=E_{s}L$, with $E_{s}$ being the electric field in the channel that produces the saturation velocity [48].

## 3. Results

**Figure 5:**
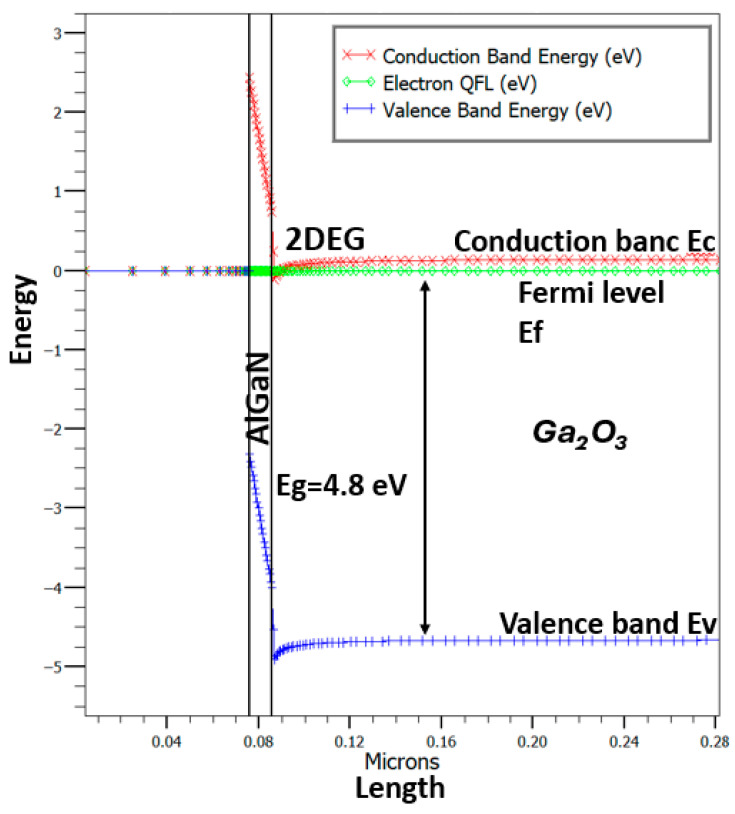
Ga_2_O_3_ HEMT band diagram of the heterostructure at zero bias conditions (at V_g_ and V_d_ = 0 V) without foreign substrate, with Q_f_ = 1 × 10^10^ cm^−2^. The downward bands indicating to the 2DEG confirmed close to the interface.

In this work, the DC operation of an n-type Ga_2_O_3_ HEMT device was simulated using Silvaco TCAD 2D device software. A 200 nm Ga_2_O_3_ buffer layer was placed from the source to drain beneath the gate to establish a conductive channel and enable normally-on operation ($V_{th}<$ 0). Figure 2 shows the device structure, highlighting a high electron concentration at the interface, confirming the presence of a two-dimensional electron gas (2DEG), as shown in Figure 3. In the positive surface charge model (Qf = 1 × 10^10^ cm^−2^), the 2DEG is formed near the β-Ga_2_O_3_ interface due to dipole bonds induced by surface charge. The peak electron concentration reaches approximately 5 × 10^20^ cm^−3^, which corresponds to a sheet carrier density on the order of 10^13^ cm^−2^ [1]. The band diagram shows downward bending of the conduction band, with the Fermi level positioned near the conduction band minimum due to n-type doping, as shown in Figure 5.

**Figure 6:**
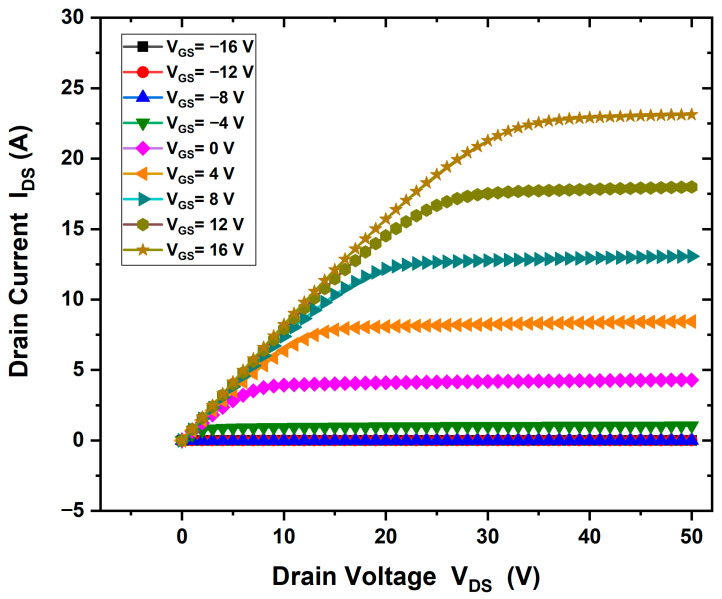
$I_{DS}-V_{Ds}$ output characteristics of the β-Ga_2_O_3_ HEMT device without substrate, gate bias of +16 V and a drain voltage of 50 V, ($I_{DS}$ = 23 A/mm).

**Figure 7:**
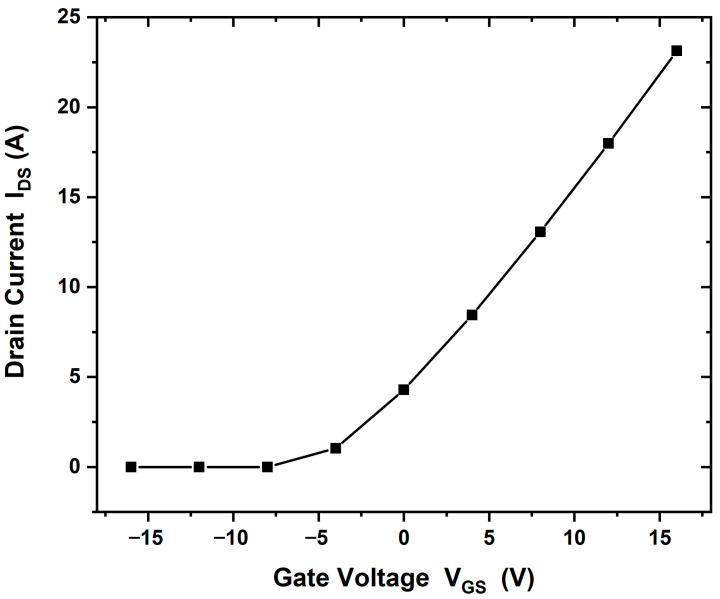
$I_{DS}-V_{Gs}$ output characteristics of the β-Ga_2_O_3_ HEMT device without substrate; threshold voltage is measured at ($V_{th}$ = −7 V).

The I–V output characteristics of the HEMT device demonstrate full channel pinch-off and current saturation ($I_{DS}$ = 23 A/mm) when a gate bias of +16 V and a drain voltage of 50 V are applied, with a voltage step of 4 V, as shown in Figure 6. The threshold voltage is measured at ($V_{th}$ = −7 V), indicating depletion-mode (normally-on) operation (Figure 7).

**Figure 8:**
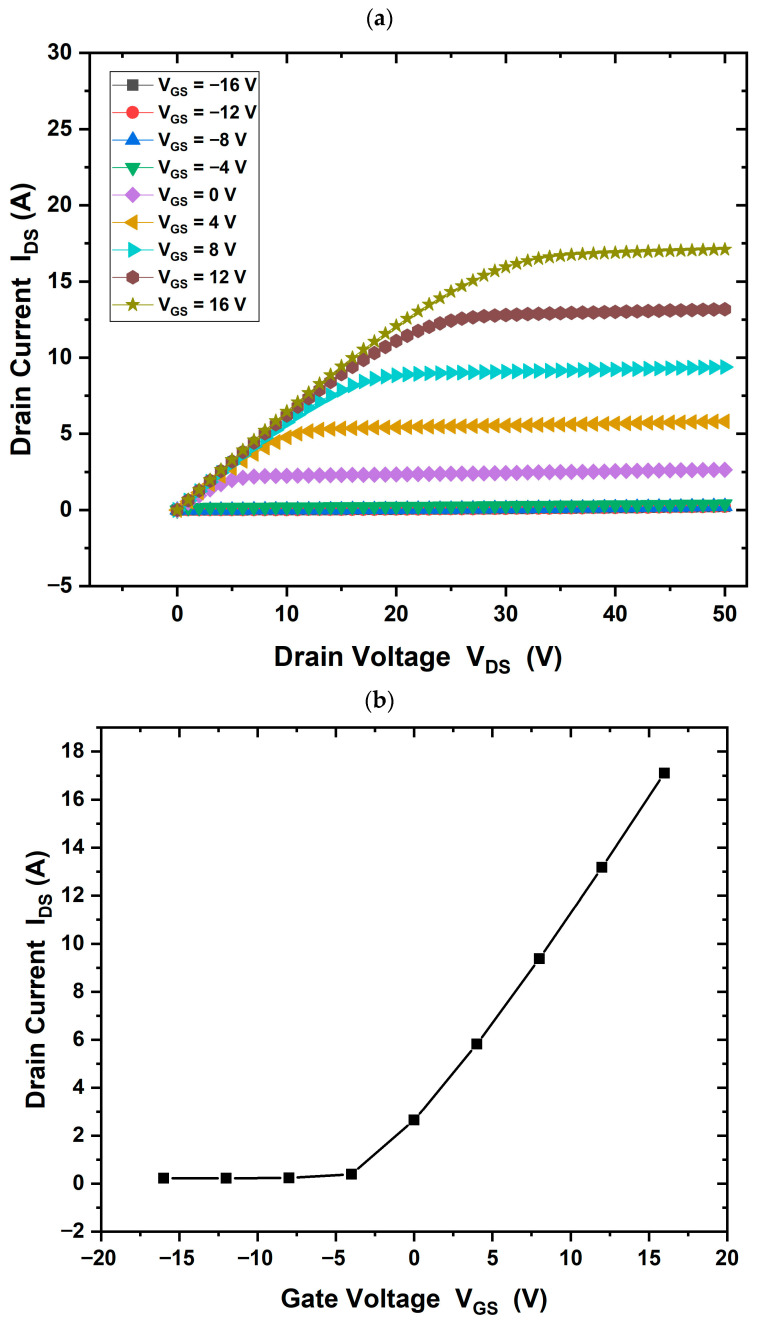
(**a**) $I_{DS}-V_{Ds}$ output characteristics of the β-Ga_2_O_3_ HEMT device with GaN substrate, gate bias of +16 V and a drain voltage of 50 V, ($I_{DS}$ = 16 A/mm). (**b**) The threshold voltage is measured at ($V_{th}$= −4 V).

**Figure 9:**
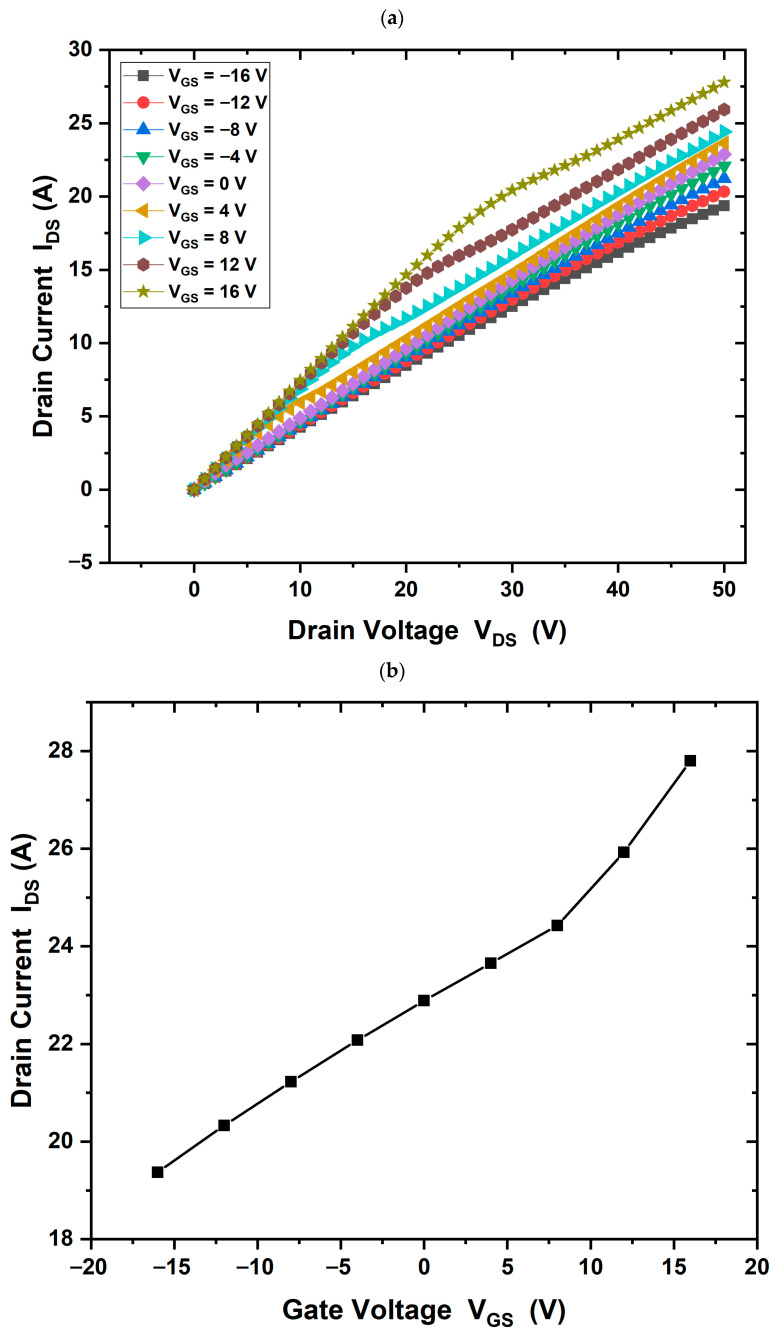
(**a**) $I_{DS}-V_{Ds}$ output characteristics of the β-Ga_2_O_3_ HEMT device with SiC substrate, gate bias of +16 V and a drain voltage of 50 V, ($I_{DS}$ = 28 A/mm). (**b**) The threshold voltage is measured at ($V_{th}$ = −16 V).

When using a GaN substrate, the drain current in the saturation region increased to ($I_{DS}$ = 16 A/mm), as shown in Figure 8a. In addition, Figure 8b shows the threshold voltage in this case ($V_{th}$ = −4 V). Using a SiC substrate, the drain current increased further to ($I_{DS}$ = 28 A/mm) and the threshold voltage was measured at ($V_{th}$ = −16 V), as shown in Figure 9a,b. This enhancement may be attributed to the electric field generated at the Ga_2_O_3_/SiC interface.

## 4. Discussion

In electronic devices, interface polarization typically refers to the buildup of electric charge or the alignment of dipole bonds at the interface between two materials. This phenomenon is common in heterojunction devices, such as metal/semiconductor or semiconductor/semiconductor structures. In this work, the Ga_2_O_3_ buffer layer was n-type-doped, and the GaN substrate was assumed to be p-type. This heterojunction allows for spontaneous or piezoelectric polarization to occur due to lattice mismatch or crystal structure differences.

Interface dipoles are formed by charge transfer, leading to carrier accumulation—specifically, the formation of a 2DEG in the HEMT structure. Assuming the GaN substrate has a p-type doping concentration of 1 × 10^17^ cm^−3^ and the Ga_2_O_3_ buffer layer has an n-type doping concentration of 1 × 10^18^ cm^−3^, the Fermi level is pinned near 0.2 eV below the conduction band minimum, as shown in Figure 5. When a positive fixed sheet charge is applied at the interface, it creates an electric field that shifts the Fermi level closer to the conduction band. As the barrier width increases, the surface donor level approaches the Fermi level, allowing electrons to transfer from surface donor states into the conduction band. Simultaneously, a conduction band offset (ΔEc = 0.95 eV, Figure 5) at the heterojunction creates an energy step that enables polarization-induced electron accumulation at the interface, leading to 2DEG formation.

On β-Ga_2_O_3_, the conduction band intersects downward with the Fermi level and forms a triangular quantum well, capable of holding a significant number of trapped electrons. In contrast, a negative sheet charge at the AlGaN/β-Ga_2_O_3_ interface fails to induce 2DEG formation.

Current–voltage characteristics:

The current–voltage (I–V) characteristics of the simulated HEMT were analyzed using the fixed positive interface charge model, as discussed in the Results section. In the baseline structure (without GaN or SiC substrate), the simulation shows high current density when a positive gate voltage is applied to the n-type Ga_2_O_3_ channel. As shown in Figure 6, the drain current reaches a maximum of 23 A/mm at a gate bias of +16 V, with a channel length of 9 µm (L_GD_ = 3.5 µm) and a gate length of 2 µm. The threshold voltage ($V_{th}$ = −7 V) confirms normally-on operation, as shown in Figure 7. The onset gate voltage (threshold) in a device like an HEMT corresponds to the semiconductor bandgap. The wide-bandgap semiconductors often allow for higher V_th_ devices due to higher breakdown strength, different charge distributions, and so on. It reflects the condition where enough charge is accumulated at the interface to confirm a conductive layer. The channel starts to conduct when applied gate bias. It depends on many factors such as the work function difference between the gate metal and semiconductor, interface charges (fixed or polarization charges), and the doping concentration.

The enhancement mode behavior is primarily enabled by interface dipole polarization, which establishes the 2DEG and modulates channel conductivity. Deep donor doping in Ga_2_O_3_ (1 × 10^18^ cm^−3^) was used to fix the Fermi level in a position favorable for channel inversion. This is supported by Figure 5, where the device characteristics confirm enhancement-mode operation. These results align with previous experimental studies, such as those in [7].

In alternate structures with GaN and SiC substrates, similar I–V characteristics were obtained. Figure 8 and Figure 9 show comparative simulation results of the drain current. The maximum drain current value is different between GaN and SiC substrates that applied with a gallium oxide layer, possibly due to lattice mismatch or crystallographic incompatibility. The drain current values obtained were Ga_2_O_3_, 23 A/mm; with GaN substrate, 16 A/mm; and with SiC substrate, 28 A/mm, respectively. Ga_2_O_3_ is monoclinic, while GaN is hexagonal, making direct epitaxial growth difficult. The literature suggests the use of a transition layer between the buffer and substrate to address this issue [8,12]. Although GaN had minimal effect on performance, SiC substrate significantly improved the HEMT operation.

In summary, as confirmed in the results section, the β-Ga_2_O_3_-based HEMT demonstrates stable and high-performance operation in normally-on mode. These findings suggest that Ga_2_O_3_ is a strong candidate for next-generation power semiconductor applications due to its favorable electronic properties and cost-effectiveness compared to other UWBG materials. It has low thermal conductivity, which is much worse in SiC and GaN for heat removal. This can lead to hot spots, self-heating, and reliability issues under high current/high power [49]. Moreover, the possibility of growing large, high-quality, native Ga_2_O_3_ bulk crystals can reduce costs compared to heteroepitaxially growing GaN or SiC or using foreign substrates [50].

## 5. Conclusions

A computational study on the effect of a Ga_2_O_3_ buffer layer with a positive fixed surface charge model in an HEMT structure was presented in this paper. The device was designed using TCAD simulations, with gallium oxide grown on a gallium nitride (GaN) substrate. The modeling process began by defining the β-Ga_2_O_3_ material and device parameters in the simulation environment. The results demonstrate that a high-density two-dimensional electron gas (2DEG) can be formed in the Ga_2_O_3_ HEMT structure, which significantly contributes to improved output characteristics, including high drain current. In addition, a relatively high threshold voltage was observed, confirming the device’s normally-on operation and indicating enhanced performance due to strong interface polarization effects. These promising results support the potential of β-Ga_2_O_3_ as a next-generation material for power electronic devices. Its advantages in polarization behavior, current handling capability, and compatibility with GaN or SiC-based substrates make it a competitive alternative to other wide-bandgap (WBG) materials.

## Figures and Tables

**Table 1:** Detailed structural parameters of the HEMT device.

Layer	Material	Thickness (nm)	Doping Concentration (cm^−3^)
Barrier layer	AlGaN	10	Undoped
Buffer layer	β-Ga_2_O_3_	200	n-type 1 × 10^18^ cm^−3^
Substrate	GaN	1200	p-type 1 × 10^17^ cm^−3^
Substrate	SiC	1200	Undoped

**Table 2:** Material parameters values incorporated into the simulation process [47].

Parameter	Symbol	Unit	Value
β-Ga_2_O_3_ Bandgap	E_g_	eV	4.8
Permittivity	ɛ_r_	-	10
Effective conduction band density of state	nc300	cm^−3^	3.72 × 10^18^
Effective valence band density of state	nv300	cm^−3^	3.72 × 10^18^
Electron mobility in the surface region	μ_n_	${\mathrm{cm}}^{2}V\cdot$s	118 cm^−2^/Vs
Hole mobility in the surface region	μ_p_	${\mathrm{cm}}^{2}V\cdot$s	50 cm^−2^/Vs
Electron affinity	E_A_	eV	4.0
Contact gate work function	W_Fg_	eV	5
Contact drain and source	W_Fd_	eV	3.93
Interface sheet charge Q_f_ (positive)	Q_f_	cm^−2^	1 × 10^10^

**Table 3:** Structural details for device simulation.

Structural Aspect	Included	How It Is Modelled in Silvaco TCAD
Fixed interface charge	Yes	interface with qf = 1 × 10^10^
Polarization and strain	Yes	model polarization calc.strain
Basic material structure	Yes	custom material definitions
SRH recombination	Yes	models srh
Carrier mobility	Partial	tmun
Traps/defects	Yes	maxtrap = 20
Interface traps	Yes	defined
Thermal effects	Yes	low thermal conductivity input for Ga_2_O_3_

## Data Availability

The original contributions presented in this study are included in the article. Further inquiries can be directed to the corresponding authors.
